# evolSOM: An R package for analyzing conservation and displacement of biological variables with self-organizing maps

**DOI:** 10.1093/bioadv/vbae124

**Published:** 2024-08-22

**Authors:** Santiago Prochetto, Renata Reinheimer, Georgina Stegmayer

**Affiliations:** Instituto de Agrobiotecnología del Litoral, FBCB-UNL, Universidad Nacional del Litoral, CONICET, CCT-Santa Fe, Santa Fe, 3000, Argentina; Research Institute for Signals, Systems and Computational Intelligence, sinc(i), FICH-UNL, CONICET, CCT-Santa Fe, Santa Fe, 3000, Argentina; Instituto de Agrobiotecnología del Litoral, FBCB-UNL, Universidad Nacional del Litoral, CONICET, CCT-Santa Fe, Santa Fe, 3000, Argentina; Research Institute for Signals, Systems and Computational Intelligence, sinc(i), FICH-UNL, CONICET, CCT-Santa Fe, Santa Fe, 3000, Argentina

## Abstract

**Motivation:**

Unraveling the connection between genes and traits is crucial for solving many biological puzzles. Ribonucleic acid molecules and proteins, derived from these genetic instructions, play crucial roles in shaping cell structures, influencing reactions, and guiding behavior. This fundamental biological principle links genetic makeup to observable traits, but integrating and extracting meaningful relationships from this complex, multimodal data present a significant challenge.

**Results:**

We introduce evolSOM, a novel R package that allows exploring and visualizing the conservation or displacement of biological variables, easing the integration of phenotypic and genotypic attributes. It enables the projection of multi-dimensional expression profiles onto interpretable two-dimensional grids, aiding in the identification of conserved or displaced genes/phenotypes across multiple conditions. Variables displaced together suggest membership to the same regulatory network, where the nature of the displacement may hold biological significance. The conservation or displacement of variables is automatically calculated and graphically presented by evolSOM. Its user-friendly interface and visualization capabilities enhance the accessibility of complex network analyses.

**Availability and implementation:**

The package is open-source under the GPL (≥3) and is available at https://github.com/sanprochetto/evolSOM, along with a step-by-step vignette and a full example dataset that can be accessed at https://github.com/sanprochetto/evolSOM/tree/main/inst/extdata.

## 1 Introduction

In recent years, there has been a notable surge in technological advancements in the life sciences field. Researchers now have access to extensive biobanks and can collect vast amounts of multimodal data, including data from omics studies across multiple organisms, which is crucial for understanding evolution. Central to this understanding is the unraveling of the connection between genes and traits, an essential endeavor for solving various biological puzzles.

One of the key challenges of this new era is the development of robust tools capable of integrating diverse data types like biological variables (for example genes, phenotypes, among others) to uncover hidden patterns and relationships among heterogeneous sources. Self-organizing maps (SOM) have gained interest among scientists due to their ability to integrate and visualize heterogeneous data through unsupervised learning (Stegmayer et al. 2012; Betts et al. 2019; Mohnike et al. 2022). They can represent complex high-dimensional input patterns into a simpler low-dimensional discrete map, with prototype vectors (neurons) that can be visualized in a two-dimensional lattice structure and which preserve the proximity relationships of the original samples (Kohonen 2001). By examining each data point individually, and clustering variables with highly similar expression patterns along measurements, SOM enable an individualized analysis of each biological variable rather than a global one. In particular, SOM excel at capturing non-linear relationships and mapping data onto visually intuitive grids, allowing researchers to not only identify general trends but also explore particular differences within individual variables.

In this application note, we present evolSOM, a novel tool based on unsupervised machine learning that facilitates the discovery of variations between multimodal data and offers an intuitive way to visualize hidden patterns within complex data matrices. The package facilitates the integration of diverse phenotypic data types, such as morphological data or metabolomics, enabling the exploration of potential gene drivers underlying observed phenotypic changes. Specifically, evolSOM clusters biological variables (genes and/or phenotypic traits) based on their expression patterns along several conditions and maps these variables onto a reference SOM, allowing the analysis of conservation and/or displacement between conditions.

Variables conserved or displaced together may suggest membership in the same regulatory network, and the nature of the displacement itself (early/delayed/flip) may hold biological significance. In the case of variable displacements, those displacements are automatically calculated and graphically presented by the package, enabling efficient comparison. Its user-friendly interface and visualization capabilities enhance the accessibility of complex network analyses. As an illustrative example, we employed evolSOM to study the displacement of genes and phenotypic traits, successfully identifying potential drivers of phenotypic differentiation in grass leaves (Prochetto et al. 2024).

A few packages provide some of this functionality, but completely separately and not integrated into a single pipeline. For instance, the R package WGCNA (Langfelder and Horvath 2008) utilizes a correlation-based approach to depict and visualize networks of data points clustered into co-expressed “modules.” However, it lacks a mechanism for mapping test condition data onto existing modules. Furthermore, automatic calculation of the displacement or conservation of genes/phenotypes among modules is not provided. In contrast, classical clustering algorithms like k-means and hierarchical clustering (Xu and Wunsch 2009) could be employed to generate clusters with control or reference data and another clustering solution with test data. The problem arises from the inability to calculate the specific displacement of biological variables from one cluster solution to the other one because there is no inherent relation among the clusters obtained.

To the best of our knowledge, a singular package that streamlines this process in a straightforward manner is currently unavailable. The key of evolSOM lies in the presence of a control condition, serving as the foundation for clusters that integrate multimodal data. Then, the biological variables of a test condition are mapped into these established control clusters. Finally, the pipeline automatically identifies and calculates various displacement types or conservation of the biological variables, all achieved through a few automated steps in a simple manner.

## 2 The evolSOM R package

**Figure 1.**
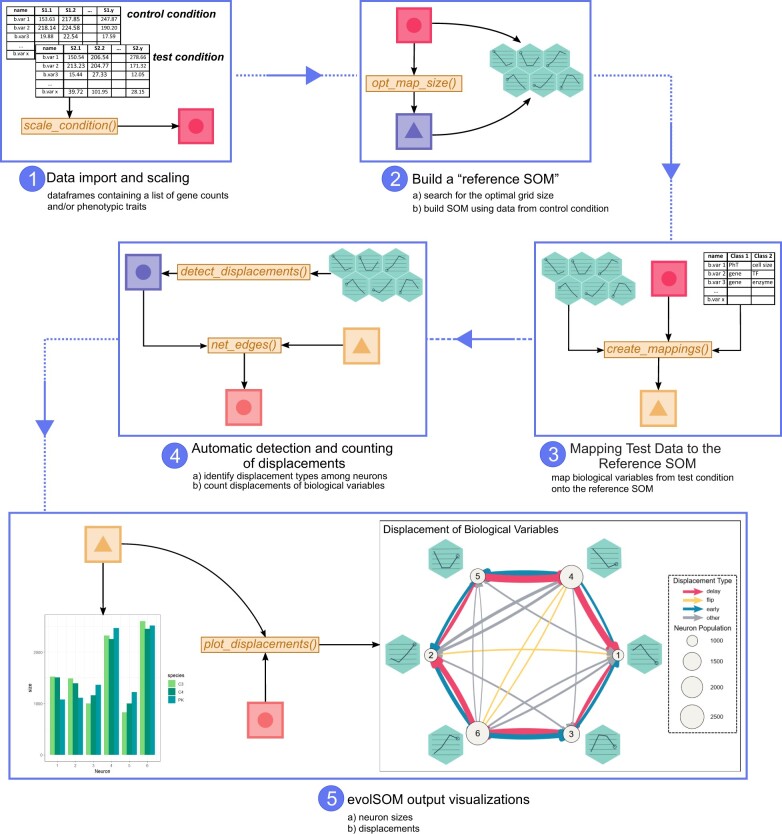
Workflow using evolSOM R package.

The package analyzes multimodal data through a series of automated steps outlined in Figure 1. Initially, pre-processed data, such as gene counts, protein levels, and anatomical measures, are imported and scaled to accommodate diverse data types (Step 1). Following this, a “reference” SOM is constructed for a control condition, optimizing the map size for efficiency (Step 2). Subsequently, biological variables from additional “test” conditions are mapped onto the reference SOM (Step 3), allowing for the assessment of conservation and/or displacement of the biological variables based on their respective neuron (cluster) locations. In the case of variable displacements, the package can automatically identify their nature (early/delayed/flip) and quantify their overall occurrence (Step 4). Finally, it generates visualizations to emphasize the relative abundance and location of biological variables on the map and provides detailed insights (Step 5). The following sections explain in detail and with examples every step of the evolSOM package workflow.

### 2.1 Data importing and scaling

The package accepts data in comma-separated value format. Rows represent biological variables (e.g., gene transcripts, phenotypes, metabolites), while columns represent measurements (e.g., tissues, cells, developmental stages, species). Users must choose a control/reference condition, and one or more test conditions according to the research question. Biological variables of interest must be measured along the same type and number of measurements in both control and test conditions to make the comparison. Data from control and test conditions are provided separately, as different dataframes. Example data frames from a practical case study are shown in Supplementary Tables S1 and S2.

Scaling the data before constructing a SOM constitutes a critical preprocessing step that enhances the performance, interpretability, and robustness of the SOM. If variables have different scales, those with larger scales dominate distance calculations, compromising accurate clustering. Scaling ensures all variables contribute equally to similarity calculations and enhances robustness to outliers. The scale_condition() function scales data to unit variance per row, i.e. (value—mean value)/standard deviation, for each biological variable separately, returning a single list containing one data frame per condition. Example outputs from the case study are shown in Supplementary Table S3.

### 2.2 Building a “reference SOM”

In order to illustrate the conservation/displacement of biological variables across diverse conditions, the evolSOM package constructs a “reference SOM.” However, determining its optimal grid size is crucial. This optimal size ensures the faithful representation of all expression patterns by neurons, ensuring each pattern’s uniqueness. Manual determination of the best grid size can be challenging, particularly for those less familiar with SOM, because it depends not only on the number of biological variables under analysis but also on the conditions under which the data were measured.

The opt_map_size() algorithm systematically evaluates different grid sizes to encompass all non-redundant expression patterns. The resulting “map_size” defines the grid’s optimal dimensions. Illustrated in Supplementary data, this algorithm takes inputs such as the dataset *D*, the correlation threshold *ρ*, the initial map size *d*, and the number of SOM iterations *i*. The initial map size s is determined by the formula $d^{2}+1$, and the variable *X* is initialized to count neurons with correlations exceeding the threshold. The SOM dimensions *d*_1_ and *d*_2_ are then determined at the ceiling and floor of *s−*1, and multiple SOM are iteratively built with dimensions *d*_1_ and *d*_2_, trained with dataset *D*. For each trained map, pairwise correlations $\rho_{i,j}$ of all map neurons are calculated and checked against the correlation threshold *θ*. The number of neurons exceeding *θ* is recorded. A null count indicates that all neurons are above the correlation threshold, ensuring an optimal distribution of data samples across the map. When this condition is fulfilled, the optimum SOM map dimensions are returned. With the determined optimal SOM map dimensions, the reference SOM is constructed and visualized using the aweSOMv1.3 library (https://cran.r-project.org/web/packages/aweSOM).

### 2.3 Mapping test data to the reference SOM

After building the “reference SOM,” the data from the test condition are mapped into the “reference SOM” to calculate conservation/displacements with the create_mappings() function. Along with the reference SOM object, the inputs of this function include the test and control condition data. Additionally, an extra dataframe is incorporated to provide supplementary categorical information about each variable, such as its type (gene or phenotypic trait), involvement in biological processes, gene family affiliation, and more (Supplementary Table S4). The mappings object stores valuable information about the mappings, such as class assignments and number of features allocated to each neuron. The results include a “class dataframe,” indicating the original neuron location in the reference SOM for each biological variable, along with its new location after mapping (Supplementary Table S5).

### 2.4 Automatic detection and counting of conservation and displacements

To uncover hidden patterns between the test and the reference condition, the package employs the detect_displacements() function to identify movements or displacements among conditions. This function scrutinizes relationships between expression patterns of neurons in the reference SOM, categorizing these relationships into four types: early, delay, flip, and other.

The discovery of conservation or displacement of biological variables involves, essentially, the cross-correlation between two data series. The data series can be temporal (i.e. different times of an experiment, or tissues with a differential degree of development) or not temporal (i.e. different tissues or organs). If the cross-correlation is maximum at the middle point of the data series, i.e. the two series are identical, conservation is observed. When the correlation is maximum at the right part of the middle point of the series and surpasses the delay_threshold, it indicates a delay displacement, which means a delay in the expression of the biological variable under analysis in the test condition with respect to the control condition. Similarly, if the correlation is maximum at the left part of the middle point of the series, it indicates an early displacement (early expression in test with respect to control condition). Conversely, a negative cross-correlation lower than the flip_threshold indicates a flip, i.e. a completely opposite behavior in the test condition with respect to the reference one. While the terms “early” and “delay” might sound as time-related, in non-temporal data they represent a shift in the sequence of activation of a biological variable compared to the reference condition and it will hardly depend on the particular order of the measurements in the data series chosen by the user. The measurements might not necessarily imply a time difference, but rather a difference in the underlying process governing the biological variables expression. The displacement object provides information about the detected displacement types (if there are some), facilitating a comprehensive exploration of displacement patterns between conditions. Then the net_edges() function, using the displacement types and the mapping object, creates a dataframe detailing the number of displacements between neurons and conditions pairwise (Supplementary Table S6). This output facilitates analysis of prevalent displacement types, or identification of broader trends, among others.

### 2.5 evolSOM output visualizations

Using information from the create_mapping() output, a bar graph can be plotted to illustrate the allocation of biological variables to each neuron in both the reference SOM and the test conditions. The output from create_mapping() and net_edges() is instrumental in plotting a displacement graph using the plot_displacements() function. This visualization represents neurons as circles, with sizes corresponding to the number of variables allocated to them. Displacements are depicted as arrows connecting neurons, the lengths of which are proportional to the number of variables displaced. Additionally, by selecting a specific group of biological variables, researchers can explore whether a distinct displacement pattern exists for this particular group. This offers valuable insights into the dynamics of this group of variables and their relationships between the reference condition and the test condition.

## 3 *C*_4_ photosynthesis as a case study

To exemplify evolSOM’s power in dissecting gene–trait relationships, we revisit a prior study (Prochetto et al. 2024) focusing on *C*_4_ photosynthesis. In that work, we employed evolSOM to shed light on leaf anatomy differentiation and evolution in grass species with different photosynthetic pathways (*C*_3_ and *C*_4_). Combining leaf transcriptome data representing different developmental stages (Prochetto et al. 2023) with detailed anatomical measurements, we aimed to identify the genetic underpinnings of this lineage leaf structural diversity. We constructed a reference SOM utilizing data from the *C*_3_ species, and subsequently, we mapped the data from *C*_4_ onto this reference SOM for further analysis. Our analysis centered on bundle sheath (BS) cell size, a crucial phenotypic trait in *C*_4_ anatomy. Using evolSOM, we identified 41 genes with expression patterns intricately linked to BS cell size across *C*_3_ and *C*_4_ grass species. Among the displaced genes there were two known components of suberin biosynthesis, a characteristic polymer in *C*_4_ bundle sheath cell walls and two displaced transcription factors, suggesting potential regulatory mechanisms underlying the observed phenotypic shift. This case study demonstrates evolSOM’s ability to unveil expression displacement patterns linked to adaptation and identify key biological processes and candidate regulatory genes associated with complex evolutionary transitions. The data frames of this practical case study can be found in the Supplementary data, Tables S1–S6. This example dataset can be freely accessed at github.com/sanprochetto/evolSOM/tree/main/inst/extdata.

## 4 Conclusions

Our package represents a valuable contribution to biologists by offering a powerful toolset for analyzing, visualizing, and interpreting complex multimodal data. By integrating genotypic and phenotypic attributes, evolSOM provides a valuable resource for unveiling intricate relationships within biological systems. Its distinct ability to map data onto SOM and analyze conservation and or displacement patterns facilitates a nuanced exploration of expression patterns dynamics across conditions. Beyond its primary applications outlined in the preceding sections, the evolSOM package holds versatile potential for other scientific investigations. The package could find utility in the exploration of temporal dynamics within biological systems, enabling the identification of temporal shifts in gene expression or phenotypic traits. Similarly, it can be applied to investigate spatial dynamics within organisms or analyze knockdown or overexpression lines alongside wild-type data in genetic experiments. Furthermore, evolSOM may contribute to understanding inter-individual variability by analyzing and comparing diverse datasets, such as omics data from individuals. Its user-friendly interface and visualization capabilities make it accessible to a broad audience, extending its utility beyond bioinformatics experts to researchers across various domains interested in exploring complex relationships within multi-dimensional datasets.

## Supplementary Material

vbae124_Supplementary_Data

## Data Availability

The package is open-source under the GPL (≥3) and is available at https://github.com/sanprochetto/evolSOM, along with a step-by-step vignette and a full example dataset that can be accessed at https://github.com/sanprochetto/evolSOM/tree/main/inst/extdata.
