# Supplementary material for: evolSOM: An R package for analyzing conservation and displacement of biological variables with self-organizing maps
Source: Bioinform Adv. 2024 Aug 22;4(1):vbae124. doi: 10.1093/bioadv/vbae124 (PMC11361812; doi:10.1093/bioadv/vbae124)
Supplement: vbae124_Supplementary_Data [file vbae124_supplementary_data.pdf]

Supplementary Material

evolSOM: an R Package for analyzing conservation and displacement of biological variables with Self-Organizing Maps

Santiago Prochetto[1,2], Renata Reinheimer[1], Georgina Stegmayer[2]

[1]Instituto de Agrobiotecnologia del Litoral, FCA-Universidad Nacional del Litoral, CONICET, CCT-Santa Fe, Ruta Nacional N° 168 Km 0, s/n, Paraje el Pozo, 3000, Santa Fe, Argentina

[2]Research Institute for Signals, Systems and Computational Intelligence, sinc(i), FICH-UNL, CONICET,CCT-Santa Fe, Ruta Nacional N° 168 Km 0, s/n, Paraje el Pozo, 3000, Santa Fe, Argentina

Table S1: **Example of input C_3 data (reference data) for a practical case using evolSOM**. Rows represent biological variables (phenotypic traits and gene transcripts), while columns represent measurements (four leaf segments along the leaf: segment 1, segment 3, segment 5 and segment 7 (for more details see Prochetto et al., 2024). The reference condition is data from C_3 species (Table S1), while the test condition is data from C_4 species (Table S2). Number and type of leaf segments must be homologous between species for effective comparison. Each treatment condition (in this case, species), are loaded as separate dataframes.

**Biological Variable**	**C3_Segment1**	**C3_Segment3**	**C3_Segment5**	**C3_Segment7**
**VB Distance**	153.6289	217.8502	202.994	201.727
**VB Density**	16.2442968	13.7412794	17.0657991	27.9270588
**IBS Cell size**	63.84	66.806	67.0657375	58.742
**OBS Cell size**	1166.38773	1563.14776	1437.66651	732.56787
**M Cell size**	218.136	224.58	242.668	190.848
**Leaf Size**	4228877.12	4878107.14	3930011.27	1737740.27
**1 VB Density**	4.1491185	3.18958832	3.62190325	5.77252106
**OG0000001**	36.5360281	42.6292532	31.7974545	88.2934568
**OG0000002**	10.4168754	12.440006	12.5414381	19.7129437
...				

Table S2: **Example of input C_4 data (test data) for a practical case using evolSOM**. Rows represent biological variables while columns represent measurements. This table shows the data from the test condition C_4 species, when the reference condition is data from C_3 species (Table S1).

Biological Variable	C4_Segment1	C4_Segment3	C4_Segment5	C4_Segment7
**VB Distance**	182.682	114.468964	100.149	100.126
**VB Density**	47.267665	64.3698648	71.0772767	71.7785461
**IBS Cell size**	135.413072	181.347258	188.982904	194.539523
**OBS Cell size**	0	0	0	0
**M Cell size**	357.102	418.408	381.49	370.94
**Leaf Size**	338685.85	508709.31	523725.3	403095.6
**1 VB Density**	19.206	11.771	11.245	10.312
**OG0000001**	115.545908	115.655958	108.352367	165.12605
**OG0000002**	55.2788222	59.8615382	52.4358709	90.1476015
**. . .**				

Table S3: **Example of output from scale_condition() using C_3 data (Table S1)**. Rows represent biological variables (phenotypic traits and gene transcripts), while columns represent measurements.

Biological Variable	C4_Segment1	C4_Segment3	C4_Segment5	C4_Segment7
**VB Distance**	-1.46101	0.806484	0.347599	0.306929
**VB Density**	-0.35042	-0.89052	-0.19014	1.431089
**IBS Cell size**	-0.04697	0.690656	0.753707	-1.3974
**OBS Cell size**	-0.02722	0.835808	0.589132	-1.39772
**M Cell size**	-0.00529	0.285579	1.059674	-1.33997
**Leaf Size**	0.440762	0.7479	0.283141	-1.4718
**1 VB Density**	0.048478	-0.94643	-0.47265	1.370596
**OG0000001**	-0.4942	-0.15709	-0.79663	1.44792
**OG0000002**	-0.9124	-0.27181	-0.24229	1.42651
**. . .**				

Table S4: **Example of categorical information input data from a practical case using evolSOM.** Rows represent biological variables (phenotypic traits and gene transcripts), while columns represent categorical information about each variable, such as its type (gene or phenotypic trait), involvement in biological processes, gene family affiliation, or any other available information.

Bioogical Variable	Class1	Class2
**VB Distance**	PhT	vb
**VB Density**	PhT	vb
**IBS Cell size**	PhT	bs
**OBS Cell size**	PhT	bs
**M Cell size**	PhT	other
**Leaf Size**	PhT	other
**1 VB Density**	PhT	vb
**OG0000001**	cell wall development	
**OG0000002**	cell wall development	
**OG0000032**	cell wall development	
**OG0000085**	TF	MYB
**OG0000109**	cell wall development	
**. . .**		

Table S5: **Example of the "class dataframe" output from create_mappings() from a practical case using evolSOM.** Rows represent biological variables (phenotypic traits and gene transcripts), while columns represent categorical information about each variable. The first three columns contain the categorical information used as input (see Table S4). The last two columns show the class assignment of biological variables (which neuron) for each condition.

**Biological Variable**	**Class1**	**Class2**	**control condition**	**test condition**
**VB Distance**	PhT	vb	3	4
**VB Density**	PhT	vb	2	6
**IBS Cell size**	PhT	bs	1	6
**OBS Cell size**	PhT	bs	1	
**M Cell size**	PhT	other	1	3
**Leaf Size**	PhT	other	1	3
**1 VB Density**	PhT	vb	2	4
**OG0000001**			2	2
**OG0000002**			2	2
**OG0000032**	cell wall development		4	3
**OG0000085**	TF	MYB	6	6
**OG0000109**	cell wall development		1	6
**...**				

Table S6: **Example of the "network dataframe" output from net_edges()in a practical case using evolSOM**. This table counts the number of displacements between neurons and condition pairs. Each row represents an edge in the network, detailing a connection between a source node and a target node. The columns provide specific information about these connections. Source: starting node of the edge; Target: ending node of the edge; Weight: number of biological variables involved in each edge; Disp%: percentage of displacement associated with the connection calculated as (weight / biological variables in the neuron in the control condition) * 100; Disp_type: type of displacement, which can be "none"(conservation), "flip," "delay," "early," or "other."

**Source**	**Target**	**Weight**	**Disp%**	**Disp_type**
**1**	**1**	463	30.50	none
**1**	**2**	103	6.79	flip
**1**	**3**	423	27.87	delay
**1**	**4**	228	15.02	early
**1**	**5**	64	4.22	other
**1**	**6**	237	15.61	other
**2**	**1**	143	9.62	flip
**2**	**2**	369	24.83	none
**2**	**3**	76	5.11	other
**2**	**4**	404	27.19	other
**2**	**5**	226	15.21	delay
**2**	**6**	268	18.03	early
**3**	**1**	108	10.84	early
...				

Algorithm 1: **opt_map_size()**, automatic optimum SOM size determination.

Data: $D, \theta = 0.8, d = 5, i = 500$
Result: $d1, d2$

$s \leftarrow d^2 + 1$; `/* Determine initial SOM grid size */`
$x \leftarrow 1$;
while $\sum(x) > 0$ **do**
  $s \leftarrow s - 1$;
  $d1 \leftarrow \lceil \sqrt{s} \rceil$;
  $d2 \leftarrow \lfloor \sqrt{s} \rfloor$;
  $SOM \leftarrow kohonen :: som(D, d1, d2, ''hexagonal'', ''euclidean'')$;
  `/* Train SOM map */`
  $\rho_{i,j} \leftarrow corr(n_i, n_j), \forall n_i, n_j, i \neq j \in SOM$; `/* Pairwise cross-correlation among SOM map neurons */`
  $x \leftarrow \rho_{i,j} > \theta$;
return $d1, d2$
